# Impact of extramedullary multiple myeloma on outcomes with idecabtagene vicleucel

**DOI:** 10.1186/s13045-024-01555-4

**Published:** 2024-06-06

**Authors:** Saurabh Zanwar, Surbhi Sidana, Leyla Shune, Omar Castaneda Puglianini, Oren Pasvolsky, Rebecca Gonzalez, Danai Dima, Aimaz Afrough, Gurbakhash Kaur, James A. Davis, Megan Herr, Hamza Hashmi, Peter Forsberg, Douglas Sborov, Larry D. Anderson Jr, Joseph P. McGuirk, Charlotte Wagner, Alex Lieberman-Cribbin, Adriana Rossi, Ciara L. Freeman, Frederick L. Locke, Shambavi Richard, Jack Khouri, Yi Lin, Krina K. Patel, Shaji K. Kumar, Doris K. Hansen

**Affiliations:** 1 Division of Hematology, Department of Medicine, Mayo Clinic, 200 1st St SW, Rochester, MN 55905 USA; 2 Stanford University School of Medicine, Stanford, CA USA; 3 The University of Kansas Medical Center, Kansas City, KS USA; 4 Blood and Marrow Transplant and Cellular Immunotherapy, H. Lee Moffitt Cancer Center, Tampa, FL USA; 5 Department of Lymphoma/Myeloma, Division of Cancer Medicine, The University of Texas MD Anderson Cancer Center, Houston, TX USA; 6 Cleveland Clinic Taussig Cancer Center, Cleveland, OH USA; 7 UT Southwestern Harold C. Simmons Comprehensive Cancer Center, Dallas, TX USA; 8 Medical University of South Carolina, Charleston, SC USA; 9 Roswell Park Comprehensive Cancer Center, Buffalo, NY USA; 10 Memorial Sloan Kettering Cancer Center, New York, NY USA; 11 University of Colorado Anschutz Medical Campus, Aurora, CO USA; 12 The University of Utah Huntsman Cancer Institute, Salt Lake City, UT USA; 13 Department of Medicine, Hematology and Medical Oncology, Icahn School of Medicine at Mount Sinai, New York, NY USA

**Keywords:** BCMA CAR-T, Ide-cel, Relapsed/refractory myeloma, Radiation, Immunotherapy

## Abstract

Idecabtagene vicleucel (Ide-cel) has demonstrated excellent efficacy and durable responses in patients with relapsed/refractory multiple myeloma (RRMM). However, the outcomes with ide-cel in patients with extramedullary disease (EMD) remain incompletely characterized. We included patients with RRMM treated with ide-cel between May 2021 and April 2023 across 11 US academic institutions. Visceral or soft tissue lesions non-contiguous from bone was classified as EMD. Time-to-event analyses were performed from date of ide-cel infusion. Among 351 patients, 84 (24%) had EMD prior to infusion. The median follow-up from ide-cel infusion was 18.2 months (95% CI: 17-19.3). The day 90 overall response rates (ORR) were 52% vs. 82% for the EMD and non-EMD cohorts, respectively (*p* < 0.001). The median progression-free survival (PFS) was 5.3 months (95% CI: 4.1–6.9) for the EMD cohort vs. 11.1 months (95% CI: 9.2–12.6; *p* < 0.0001) for the non-EMD cohort. In a multivariable analysis, EMD was an independent predictor of inferior PFS [hazard ratio 1.5 (1.1–2.2), *p* = 0.02]. The median overall survival was 14.8 months [95% CI: 9-Not reached (NR)] vs. 26.9 months (26.3 vs. NR, *p* = 0.006) for the EMD and non-EMD cohorts, respectively. Extramedullary disease represents an independent predictor of inferior day 90 ORR and PFS among patients treated with ide-cel.

**Supplementary Information:**

The online version contains supplementary material available at 10.1186/s13045-024-01555-4.

## Introduction

Treatment options for patients with relapsed/refractory multiple myeloma (RRMM) have expanded significantly over the last decade and have resulted in improvement in overall survival (OS) for patients with multiple myeloma (MM) [1, 2]. An increasingly prevalent complication observed in RRMM is the emergence of extramedullary disease (EMD), which is associated with inferior survival outcomes independent of other well-established prognostic markers [3, 4]. Extramedullary disease can be noted in up to 2–5% patients at initial diagnosis of MM, but this prevalence rises in RRMM, where EMD can be noted in 20–40% patients [5–7]. Patients with EMD continue to have suboptimal outcomes even in the novel therapeutic era, with no discernible improvement in OS in recent years [8]. Additionally, the definition of what constitutes as EMD has evolved over the years, with current consensus being to classify patients with non-bone contiguous lesions with malignant plasma cell involvement as true EMD and bone-associated soft tissue plasmacytomas as paraskeletal disease [5, 9]. This distinction is based on consistently inferior outcomes noted with EMD compared to paraskeletal MM [10–12].

Idecabtagene vicleucel (Ide-cel), a B-cell maturation antigen (BCMA)-directed chimeric antigen receptor T-cell (CAR-T) therapy, received approval by the US food and drug administration (FDA) in March 2021 for the treatment of RRMM after exposure to at least 4 prior lines of therapy including a proteasome inhibitor, an immunomodulatory drug (IMiD) and an anti-CD38 monoclonal antibody. The single arm, phase II KarMMA trial and the subsequent randomized phase 3 KarMMA-3 trial demonstrated excellent objective response rates (ORR) of over 70% with efficacy noted across various subgroups, including patients with EMD [13, 14]. Furthermore, a progression-free survival (PFS) benefit was also demonstrated in the EMD subgroup among patients treated with ide-cel when compared to standard of care therapies in the KarMMA-3 trial [13, 14]. However, there remains a dearth of comparative data on PFS and OS with ide-cel in patients with and without EMD. Notably, the KarMMa and KarMMa-3 trials with ide-cel included paraskeletal disease within the EMD cohort, leaving the efficacy and outcomes with ide-cel in true EMD insufficiently characterized. Recent reports have highlighted inadequately sustained responses to CAR-T therapy among patients with EMD [8, 15, 16]. Given these concerns, we conducted a comprehensive evaluation of the efficacy and safety profile of ide-cel in a sizable cohort, with a particular emphasis on extramedullary disease.

## Methods

### Study cohort

The study population included patients with RRMM that were evaluable for EMD and infused with ide-cel across 11 US academic centers between May 2021 and April 2023. This study was approved by the respective institutional review boards, informed consent was obtained per respective institutional review board guidelines and the study was conducted in accordance with the Declaration of Helsinki.

### Definitions, response assessment and procedures

Response was assessed by treating investigators based on the International Myeloma Working Group (IMWG) criteria [17] but due to the retrospective nature of our study, all of the IMWG criteria were not required to be fulfilled. Patients with oligo/non-secretory disease could be assessed for complete response or progression based on immunofixation, bone marrow and imaging parameters per investigator discretion. Measurable residual disease (MRD) was determined by either flow cytometry or clonoSEQ®, per institutional practice, at a sensitivity of at least 10^− 5^ nucleated cells. Patients that died before response assessment were considered as non-responders. In addition, a PET-specific response for extramedullary sites of disease was reported, with response categories including complete response (CR), partial response (PR), stable disease (SD) and progressive disease (PD) [18]. Patients noted to have a hematologic response, but progressed on PET-CT were classified as having progression for the ORR reporting per the existing IMWG definition. High-risk cytogenetics were defined by presence of deletion 17p, t(4;14) or t(14;16)/t(14;20) at any time point prior to ide-cel infusion [19]. Extramedullary disease was defined as involvement by soft tissue or visceral lesions that were non-bone contiguous. EMD sites were broadly classified as visceral versus non-visceral, with visceral disease including patients with at least one visceral organ involvement with or without a non-visceral site of disease. Visceral sites of disease included any organ involvement. Non-visceral sites of disease included skin, soft tissue (including retroperitoneal and musculoskeletal involvement), and lymph nodes. Patients with presence of both extramedullary and paraskeletal disease (PSD) were classified as having EMD, and patients with bone-associated disease without EMD were classified as having paraskeletal disease. For patients with renal insufficiency, fludarabine dose was adjusted based on creatinine clearance per institutional protocols. Cytokine release syndrome (CRS) and immune effector cell-associated neurologic syndrome (ICANS) were graded according to American Society for Transplantation and Cellular Therapy (ASTCT) criteria [20, 21]. Hematologic toxicities were graded by NCI-Common Terminology Criteria for Adverse Events (CTCAE) version 5.0 [20, 21]. Bridging therapy was utilized at the discretion of the treating physician. Lymphodepleting chemotherapy was also determined by the treating physician and a fludarabine shortage during the study period led to a small proportion of patients receiving alternative lymphodepletion regimens instead of the standard regimen per package insert (fludarabine and cyclophosphamide).

### Statistical analysis and endpoints

The continuous variables were compared using non-parametric tests and categorical variables were compared using chi square test (or Fischer’s exact test if *n* < 15). All time-to-event analyses were performed from time of ide-cel infusion using the Kaplan Meier (KM) method and survival outcomes were compared using the log-rank test. Follow-up was calculated using reverse KM censoring. For identifying independent predictors of PFS, variables noted to be significant on a univariate analysis were included in a subsequent multivariable Cox proportional hazard analysis. All statistical analyses were performed using BlueSky Statistics©, LLC.

## Results

### Baseline characteristics

We included 351 patients infused with ide-cel in the analysis out of which, 84 (24%) were noted to have EMD prior to ide-cel infusion. Among patients without EMD, 74 patients (21% of the study cohort) were noted to have PSD. The median follow-up time from ide-cel infusion was 18.2 months (95% CI: 17-19.3 months) and the follow-up was comparable for the cohorts of patients with and without EMD [median 18.5 (95% CI: 15.8–22.1) months vs. 18.1 (95% CI: 16.7–19.9) months, respectively]. In the EMD cohort, 43% (*n* = 36) patients had visceral site of disease and 73% (*n* = 61) had more than one site of extramedullary lesion on pre-infusion imaging. The proportion of patients with high-risk cytogenetics, revised ISS stage III, triple class refractory disease and median prior lines of therapy were comparable in the two groups. Patients with EMD were younger, had a higher proportion of ECOG performance status > 1, higher baseline ferritin and CRP levels, and higher proportion of penta-drug refractory status at ide-cel infusion. The baseline characteristics for patients with and without EMD are depicted in Table 1.

**Table 1:** Comparison of clinical parameters for patients with and without extramedullary disease treated with Ide-cel

Parameter	Data available, *n* (%)	Extramedullary Disease (*n* = 84)	No Extramedullary Disease (*n* = 267)	*P* value
Age at infusion, median (IQR), years	351 (100)	62 (55–69)	66 (59–71)	**0.02**
Sex, % Females	351 (100)	38 (45)	111 (42)	
ECOG 0–1, n (%)	333 (95)	62 (78)	225 (89)	**0.02**
Revised ISS Stage III, n (%)	256 (73)	11 (23)	42 (21)	0.65
High-Risk Cytogenetics*, n (%)	308 (88)	20 (29)	79 (33)	0.47
Deletion 17p	316 (90)	16 (22)	58 (24)	0.67
t (4;14)	309 (88)	5 (7)	29 (14)	0.24
t (14;16)/t(14;20)	306 (87)	1(1)	9 (4)	0.33
1q gain/amplification	307 (87)	34 (49)	107 (45)	0.52
Prior Lines of Therapy, median (IQR)	351 (100)	6 (5–8)	6 (5–8)	0.19
Triple Class Refractory^#^, n (%)	351 (100)	74 (88)	215 (81)	0.11
Penta-drug refractory^π^, n (%)	351 (100)	39 (46)	86 (32)	**0.02**
Bridging Therapy, n %)	351 (100)	67 (80)	195 (73)	0.08
Ferritin prior to LD, median (IQR), µg/L	351 (100)	591 (326–1590)	242 (112–730)	**< 0.001**
CRP, median (IQR), mg/L	351 (100)	2.1 (2.6-9)	1 (0.3–4.6)	**0.001**
Bone marrow plasma cell burden > 50%,	323 (92)	22 (30)	69 (28)	0.66
Did not meet criteria for KarMMa1	340 (97)	65 (80)	184 (71)	0.2

IQR: interquartile range; LD: lymphodepletion; *High-risk cytogenetics defined as t(4;14), deletion 17p, t(14;16) at any time prior to lymphodepletion; ^#^ refractory to at least 1 proteasome inhibitor, 1 IMiD and a CD38 antibody; ^π^refractory to bortezomib, carfilzomib, lenalidomide, pomalidomide and either daratumumab or isatuximab

### Response rates

**Fig. 1:**
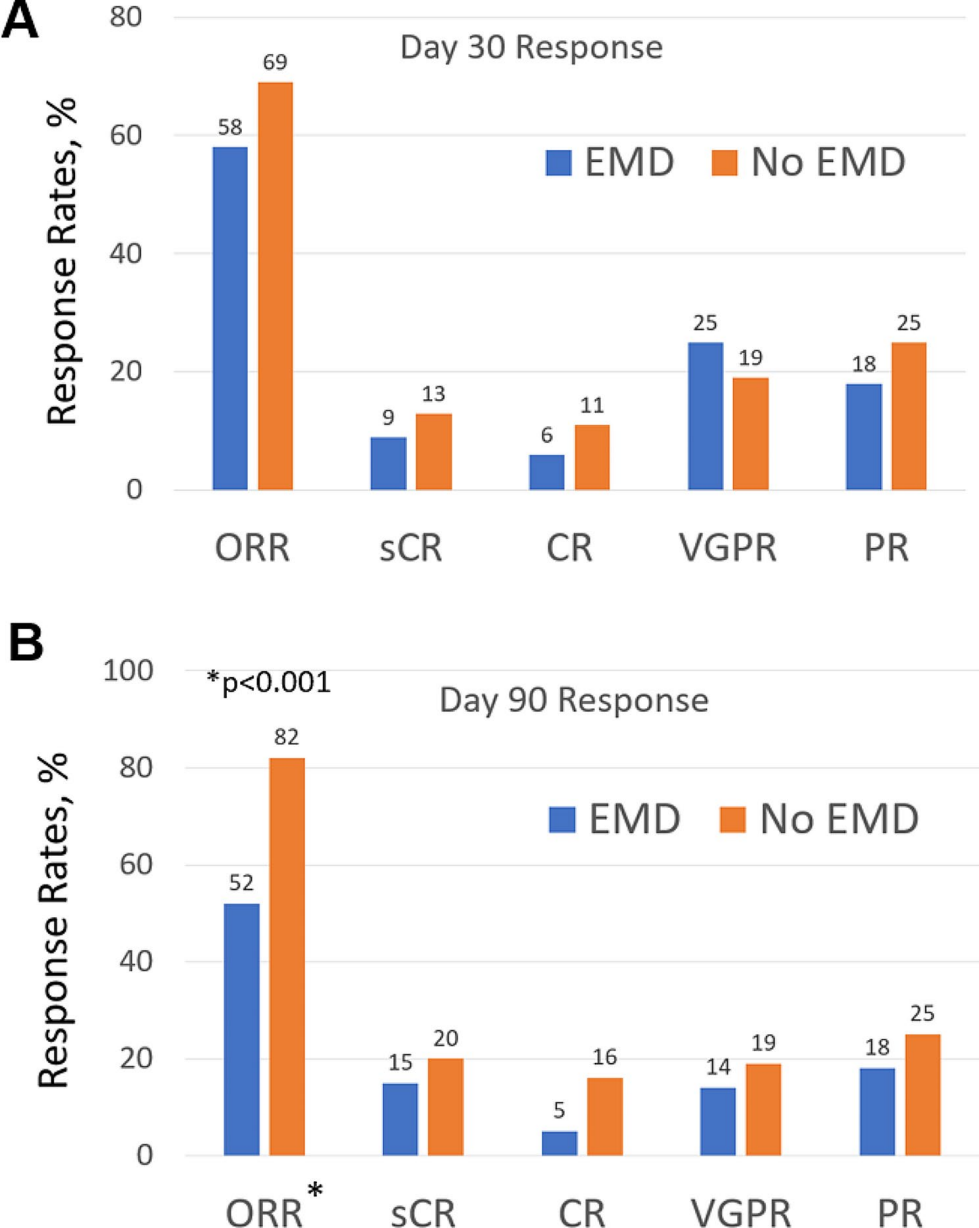
Response rates at Day 30 and Day 90 by the IMWG criteria: Patients with EMD demonstrate significantly inferior overall response rates (ORR) at day 90 (52%) compared to the non-EMD cohort (82%, *p* < 0.001)

At day 30 of ide-cel infusion, the overall response rate (PR or better) was 58% for the EMD cohort compared to 69% for the non-EMD cohort (*p* = 0.1). The rates of CR or better at day 30 were 16% (12/77) for the EMD cohort and 24% (61/253) for the non-EMD cohort (*p* = 0.11). Among patients with EMD, a PET response was available at day 30 in 45 patients (54%), out which in 24 patients (54%) achieved a PR or CR. At day 90, the ORR was 52% for EMD cohort whereas a deepening of response was noted at day 90 for the non-EMD cohort with an ORR of 82% (*p* < 0.001). Presence of EMD was an independent predictor of inferior day 90 ORR, in addition to prior BCMA-directed therapy (Supplementary Table 1). A PET response at day 90 was available in 69 (82%) patients, out of which 35 (51%) patients achieved a PR or CR. The rate of MRD negativity at day 30 (*n* = 195) was 84% for the EMD cohort and 74% for the non-EMD cohort (*p* = 0.2). At day 90 (*n* = 176), the MRD negativity rate was 60% in the EMD cohort versus 77% in the non-EMD cohort (*p* = 0.06). Rates of best ORR were 58% for the EMD cohort and 82% for the non-EMD cohort. The details of depth of response by the IMWG criteria at day 30 and day 90 are depicted in Fig. 1.

### Progression-free survival

**Fig. 2:**
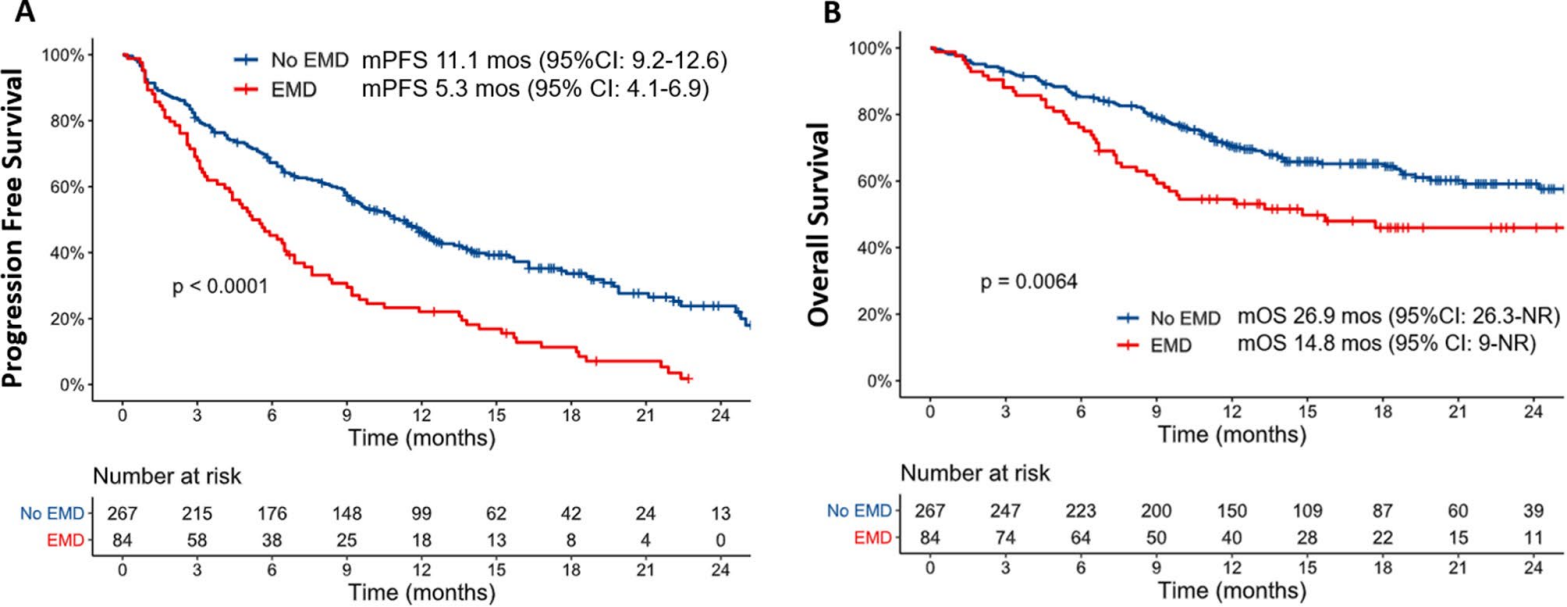
Survival Outcomes with ide-cel: **A**. Presence of extramedullary disease (EMD) was associated with a significantly inferior progression-free survival compared to the patients without EMD [hazard ratio 2.1 (95% CI: 1.6–2.7), *p* < 0.001]. **B**. Overall Survival was significantly inferior in the cohort of patients with EMD [Hazard ratio 1.6 (95% CI: 1.1–2.4), *p* = 0.007]

The median PFS for the entire cohort was 9.1 months (95% CI: 7.6–10.7) and the 18-month PFS rate was 28%. The median PFS was 5.3 months (95% CI: 4.1–6.9) for the EMD cohort vs. 11.1 months (95% CI: 9.2–12.6; *p* < 0.0001) for the non-EMD cohort (Fig. 2A). Patients with EMD demonstrated a significantly inferior PFS compared to patients with PSD [Hazard Ratio 1.7 (95% CI: 1.2–2.4), *p* = 0.005], Supplementary Fig. 1A. A univariate analysis demonstrated the presence of EMD, revised ISS Stage III, high (> 50%) bone marrow plasma cell infiltrate at lymphodepletion, use of bridging therapy, penta-drug refractory status, prior BCMA-directed therapy exposure, plasma cell leukemia, ECOG performance status > 1 and elevated pre-lymphodepletion ferritin levels (> 400 µg/L) to be associated with inferior PFS in the entire cohort (Supplementary Table 1). On a multivariable Cox regression analysis (*n* = 229), presence of EMD remained an independent marker for inferior PFS in the cohort [Hazard Ratio (HR) 1.5 (95% CI: 1.1–2.2), *p* = 0.02], Table 2.

**Table 2:** Independent predictors of inferior progression free survival an overall survival with ide-cel on multivariable analyses

Progression-free Survival (PFS)		
Parameter	PFS Hazard Ratio (95% CI)	Multivariable Analysis p-value
Extramedullary Disease	1.5 (1.1–2.2)	0.02
ECOG Performance Status ≥ 2 at LD	1.4 (0.9–2.2)	0.13
Revised ISS Stage 3 at infusion	1.2 (0.8–1.8)	0.3
Use of Bridging Therapy	1.6 (1.1–2.4)	0.02
Penta-drug refractory status	1.1 (0.8–1.6)	0.4
Prior BCMA-directed therapy exposure	1.5 (1.1–2.3)	0.03
Ferritin prior to LD > 400 µg/L	1.5 (1.1–2.2)	0.02
Plasma Cell Leukemia	2.5 (1.3–4.7)	0.008
High BPMC (> 50%) prior to LD	1 (0.7–1.4)	0.98
**Overall Survival (OS)**		
Parameter	OS Hazard Ratio (95% CI), *n* = 217	Multivariable Analysis p-value
Extramedullary Disease	1.01 (0.6–1.7)	0.95
Revised ISS Stage 3	2 (1.1–3.5)	0.02
Use of Bridging Therapy	1.8 (0.94–3.4)	0.07
ECOG Performance Status > 1 at LD	1.8 (0.95–3.4)	0.07
Serum ferritin > 400 µg/L at LD	1.8 (1.1–2.9)	0.03
≥PR at Day 90	0.3 (0.17–0.51)	< 0.001
Plasma Cell Leukemia	2.8 (1.2–6.7)	0.02
High BMPC (> 50%) at LD	0.84 (0.5–1.5)	0.55

BCMA: B-cell membrane antigen; BMPC: bone marrow plasma cells; ECOG: Eastern Cooperative Oncology Group ISS: international staging system; LD: lymphodepletion

**Fig. 3:**
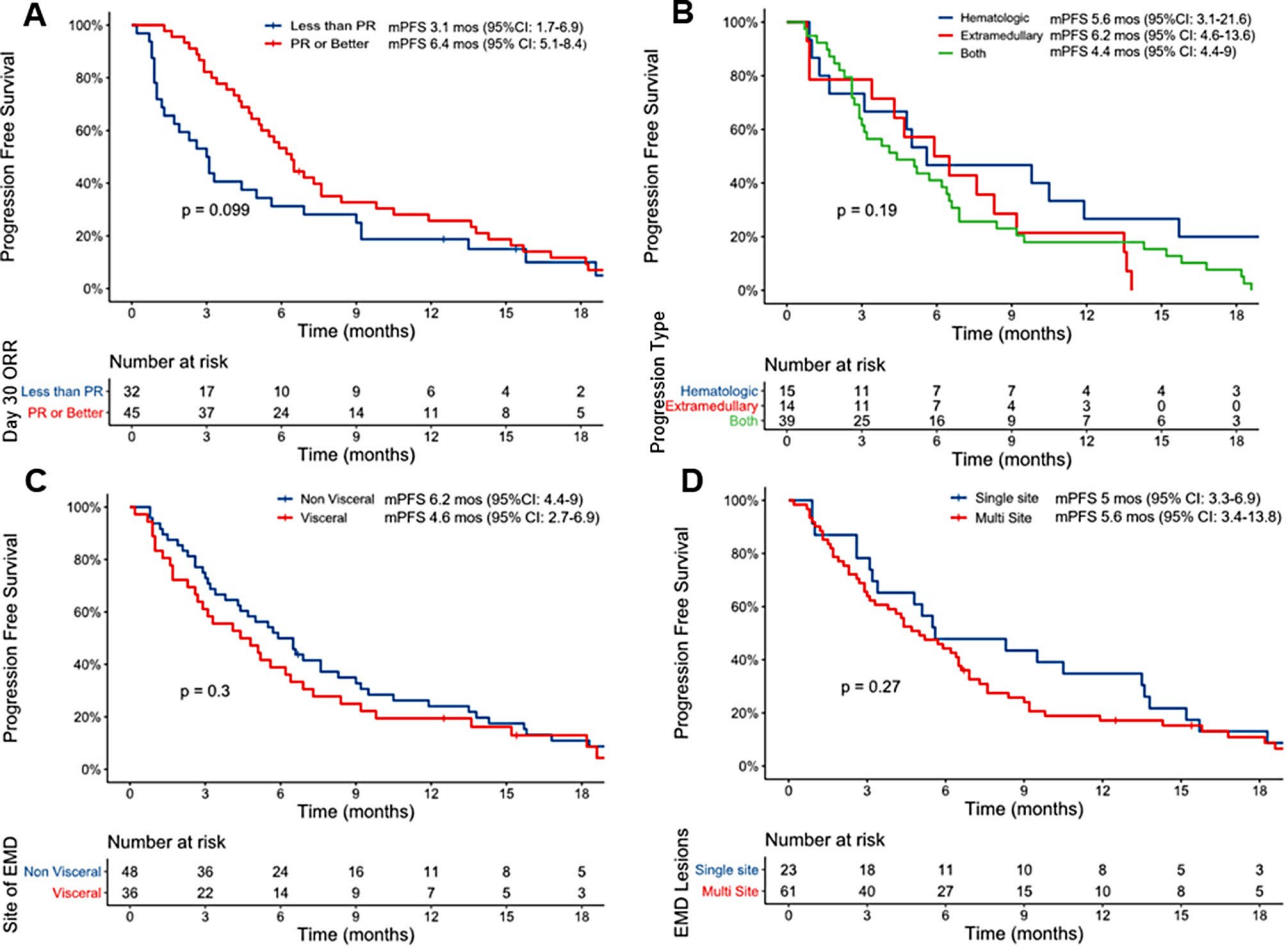
Progression-free survival (PFS) with ide-cel in patients with extramedullary disease (EMD). **A**. Patients with EMD achieving a day 30 objective response demonstrated a trend toward improved PFS but this did not reach statistical significance. **B**. The type of progression (hematologic, extramedullary or both) did not impact the PFS. **C**. Visceral site of EMD did not confer an inferior PFS with ide-cel and **D**. Presence of multi-site disease was not associated with a significantly worse PFS with ide-cel

Among patients with EMD that were evaluable for and achieved an ORR at day 30 (*n* = 77), the median PFS was 6.4 months (5.1–8.4), and the median PFS for day 30 non-responders was 3.1 months (95% CI: 1.7–6.9; *p* = 0.09, Fig. 3A). Among patients with PET-CT performed, the median PFS among day 90 among PET-CT responders (CR or PR) was 9.2 months (95% CI: 6.9–15.2) compared to 2.7 months (95% CI: 1.9–5.1) among non-responders (*p* < 0.001, Supplementary Fig. 2). Among patients with EMD that progressed (*n* = 68), both hematologic and extramedullary progression was noted in 57% (*n* = 39) patients, and isolated hematologic or extramedullary progression was noted in 22% (*n* = 15) and 21% (*n* = 14) patients, respectively. The type of progression did not impact the PFS for patients with hematologic progression only [*n* = 15, median PFS 5.6 months (95% CI: 3.1–21.6), extramedullary only [*n* = 14, median PFS 6.2 months (95% CI: 4.6–13.6)] or both hematology and extramedullary relapse [*n* = 39, median PFS 4.4 months (95% CI: 3-6.6); *p* = 0.19, Fig. 3B]. The median PFS for patients with EMD with visceral involvement was 4.6 months (95% CI: 2.7–6.9) compared to 6.2 months [(95% CI: 4.4-9) months; *p* = 0.3, Fig. 3C] for patients with non-visceral EMD. Similarly, the median PFS was comparable among patients with a single site of EMD versus multi-site EMD [median 5 months (95% CI: 3.3–6.9) versus 5.6 months (95% CI: 3.4–13.8), respectively; *p* = 0.27, Fig. 3D]. Among the patients with data available, 19 (24%) out of 79 patients received radiation therapy to any EMD site prior to ide-cel infusion. The median PFS was 6.9 months (95% CI: 5.7–13.6) for patients receiving radiation versus 4.3 months (95% CI: 3.1–6.5) without radiation prior to ide-cel (*p* = 0.77), Supplementary Fig. 3.

### Overall survival

The estimated median OS for the entire cohort 26.9 months [95% CI: 24.2-Not Reached (NR)] and the 18-month OS rate was 60%. The median OS for patients with EMD was 14.8 months (95% CI: 9-NR) and 26.9 months (95% CI: 26.3vs. NR) for the non-EMD cohort [HR 1.6 (95% CI: 1.1–2.4); *p* = 0.007, Fig. 2B]. Patients with EMD, PSD and non-EMD/non-PSD cohorts demonstrated discrepant survival outcomes (*p* = 0.002; Supplementary Fig. 1B). In a subgroup analysis restricted to the EMD and PSD cohorts, patients with EMD demonstrated numerically inferior OS [median OS 14.8 (95% CI: 9-NR)] compared to PSD [median OS 19.9 (95% CI: 14.1-NR), although this did not reach statistical significance (*p* = 0.46).

On univariate analysis, presence of EMD, revised ISS stage III, use of bridging therapy, ECOG PS > 1 at lymphodepletion and elevated serum ferritin prior to ide-cel infusion were associated with inferior OS, whereas achieving an objective response (PR or better) at day 90 was associated with an improved OS (Supplementary Table 2). On a multivariable Cox regression analysis (*n* = 217), presence of EMD was not an independent predictor of inferior OS [HR 1.01 (95% CI: 0.6–1.7), *p* = 0.9]; use of bridging therapy, revised ISS Stage III and elevated ferritin remained independent predictors of inferior prognosis and a PR or better at day 90 of ide-cel infusion was independently associated with improved OS (Table 2 and Supplementary Table 3). In the EMD cohort, presence of visceral disease [HR 1.6 (95% CI: 0.9–2.8), *p* = 0.14] and multi-site disease [HR 1.9 (95% CI: 0.9–3.9); *p* = 0.09] demonstrated a trend toward inferior OS but did not reach statistical significance (Supplementary Fig. 4). Similarly, incorporation of radiation to EMD site prior to ide-cel infusion [HR 0.44 (95% CI: 0.2–1.04), *p* = 0.056] and achieving an objective response at Day 90 [HR 0.57 (0.3–1.08), *p* = 0.08] demonstrated a trend toward improved OS for the cohort of EMD patients, but these did not reach statistical significance (Supplementary Fig. 4). Achieving an ORR at day 30 per the IMWG criteria did not impact OS [HR 0.85 (95% CI: 0.45–1.6), *p* = 0.61].

### Adverse effect profile

The rates of notable non-hematologic adverse events were comparable in the cohort of patients with and without EMD. Patients with EMD had a comparable rate of CRS [grade ≥ 2 CRS of 29% versus 22%, *p* = 0.23] and ICANS [grade ≥ 2 ICANS rate of 10% versus 8%, *p* = 0.58] in the cohort of patients with and without EMD, respectively (Supplementary Table 4). Similarly, incidence of intensive care unit hospitalization and any grade infection in the post-infusion period were comparable (Supplementary Table 4). With regard to hematologic adverse events, the rates of grade ≥ 3 neutropenia were comparable at day 30 (34% vs. 33%, *p* = 0.84), however a higher rate of grade ≥ 3 neutropenia was noted at day 90 in the cohort of patients with EMD (21% vs. 9%, *p* = 0.009). Patients with EMD had a significantly lower median hemoglobin level at day 30 (8.9 g/dL versus 9.8 g/dL, *p* = 0.003) and day 90 (9.8 g/dL versus 10.7 g/dL, *p* = 0.003) compared to the cohort of non-EMD patients, although the rates of grade ≥ 3 anemia at day 30 and day 90 were comparable (Supplementary Table 4). A trend toward higher rates of grade ≥ 3 thrombocytopenia was noted at both day 30 (54% vs. 43%) and day 90 (30% vs. 19%) for the EMD compared to non-EMD cohort (Supplementary Table 4). Comparable rates of G-CSF and thrombopoietin agonist use were noted in the two cohorts (Supplementary Table 4). Patients with EMD required a higher rate of stem cell boost (14% versus 4%, *p* = 0.001). Among the EMD and non-EMD cohort, the proportion of patients undergoing ASCT prior to lymphodepletion, pre-lymphodepletion cytopenia grades, bone marrow plasma cell burden at lymphodepletion and use of alkylators in bridging were comparable (Supplementary Table 5).

## Discussion

In this large cohort of patients with predominantly triple-class refractory RRMM treated with ide-cel, we demonstrate that presence of EMD is associated with inferior responses and a markedly reduced PFS. Extramedullary disease is evident in 10–20% of patients with RRMM and is a well-established marker of inferior prognosis, even in the era of novel therapies [22, 23]. Patients with EMD have poor responses and suboptimal PFS when treated with conventional novel agent therapies. A recent series demonstrated dismal outcomes with conventional MM-directed therapies with a median PFS of 2.2 months for proteasome inhibitor and IMiD-based combinations and 2.9 months for alkylator-based combinations [8]. Similarly, response rates and PFS with CD38-directed therapies is noted to be inferior in EMD compared to patients without EMD [23]. Immune effector therapies, including CAR-T and bispecific antibodies, have demonstrated excellent response rates ranging from 60 to 90% in heavily pretreated population of patients with MM [6, 24–27]. However, concerns persist regarding their reduced efficacy in patients with EMD [28–30]. A recent report of teclistamab use in the real-world setting also demonstrate a dismal median PFS of 2.1 months in patients with extramedullary disease, with inferior ORR and PFS for EMD also demonstrated in other series [31]. In our cohort of ide-cel treated patients, the median PFS of 5.6 months in EMD is suboptimal. However, it may still represent a more favorable option compared to many available conventional treatments.

The unique biologic aspects of EMD remain to be well-elucidated. There is a currently a dearth of information on genomic drivers that are unique to EMD. In a small study of patients with EMD at relapse, whole exome sequencing identified these tumors to be predominantly enriched in MAPK pathway mutations, which is a common feature in RRMM even without EMD [32, 33]. Transcriptionally, EMD appears to have decreased bone marrow homing through downregulation of CXCR4 [33]. A small study comparing paraskeletal and extramedullary tumors identified higher Ki-67 expression and a more immature phenotype in true EMD [34]. Additional genomic and transcriptomic studies on extramedullary tumor tissue are needed to identify unique drivers of aggressive disease and potential therapeutic targets.

Prior studies have postulated at a role of higher tumor burden leading to inferior outcomes through accelerated T-cell exhaustion [35, 36]. It is conceivable that patients with EMD possibly have a higher disease burden at CAR-T infusion than their non-EMD counterparts. Supporting this notion, we observed a decline in the ORR from day 30 to day 90 in the EMD cohort, contrasting with the deepening of responses in the non-EMD cohort, indicative of poor persistence of ide-cel in EMD. However, we did not observe worse outcomes among patients with EMD presenting with visceral or multi-site disease, suggesting the involvement of additional factors contributing to these inferior outcomes.

Recently, the role of antigen-presenting dendritic cells in regulating antigen-specific T-cell entry into the MM tumor milieu has been elucidated [37]. It is plausible that differences in the immune tumor microenvironment render immune effector therapies less effective in accessing extramedullary tumor sites, thereby leading to worse outcomes. In contrary, our finding of comparable rates of progression in both hematologic and extramedullary sites, as well as the absence of an impact of the type of progression (hematologic, extramedullary or both) on PFS, argue against poor penetration of ide-cel in the extramedullary tumor sites being the predominant reason for the inferior outcomes. A comprehensive characterization of the immune tumor microenvironment of extramedullary disease is crucial to understanding these suboptimal responses.

We observed similar rates of CRS and ICANS among the EMD and non-EMD cohorts, but the necessity for a stem cell boost was notably higher among patients with EMD. This could potentially indicate a diminished marrow reserve resulting from prior treatments in patients with EMD, although the requirement for granulocyte colony-stimulating factor (GCSF) and thrombopoietin agonists was comparable between the two cohorts. This finding is intriguing, particularly considering the comparable median prior lines of therapy exposure, rate of ASCT, rates of high bone marrow plasma cell infiltrate (> 50%) at the time of lymphodepletion, and rates of alkylator use in the bridging regimen among the two cohorts and warrants further study.

Utilizing radiation as a bridging therapy for axi-cel in patients with aggressive lymphomas demonstrated comparable adverse effect profile, favorable in-field disease control and no major impact on the feasibility of CAR-T manufacturing [38, 39]. However, there is limited data on the role of radiation therapy prior to ide-cel in RRMM. While progression events appear to have been delayed in patients with EMD who received radiation therapy prior to ide-cel infusion in our cohort (Supplementary Fig. 3), this did not reach statistical significance. There was an emerging trend toward improved OS among patients receiving radiation to EMD sites, without adjusting for other known prognostic markers. Caution is needed in interpreting these results given the small number of patients at risk in each group, but along with other small series, our findings do support feasibility of this approach [40, 41]. The other consideration is that patients with readily radio-encompassable disease may have lower disease burden and less visceral involvement than those that have disease sites not amenable to radiation, with associated bias that this could introduce.

Our study is subject to the inherent limitations of a retrospective analysis, including potential introduction of confounders. The adjudication of response in our study was performed by the investigators at respective institutions rather than being centralized, which may have resulted in non-uniformity. Additionally, the imaging was performed at the discretion of the treating centers and the lack of a standardized imaging schema for response assessment of EMD can introduce bias. In the future, it would be worthwhile to have a standardized imaging practice (e.g. with periodic PET-CTs) to aid in uniformity of response assessment. The MRD assessments included in our analysis were performed at a sensitivity of at least 10^− 5^ per individual institutional practices, and the flowcytometry-based testing conformed with the Euroflow guidelines. The day 90 MRD negativity rates demonstrated a trend toward inferiority in the EMD cohort. It is conceivable that these differences could be more pronounced with a negative MRD test with a sensitivity of 10^− 6^, which is a stronger predictor of OS in MM [42]. Although presence of EMD was an independent predictor of PFS, patients with EMD had poorer performance status and higher proportion of penta-drug refractoriness, likely impacting future treatment options and OS. Additionally, missing data and fewer events noted in the OS analysis could have precluded a comprehensive analysis of the prognostic impact of EMD on OS. A longer follow-up could potentially help ascertain this in the future. Furthermore, even among patients with EMD, approximately one-third experienced systemic-only progression, indicating an overall aggressive disease biology extending beyond the extramedullary site. The small numbers at risk in some of the subgroup analyses (e.g. analyses demonstrated in Fig. 3) may result in some small magnitude effects being missed due to low power. We consider these findings are hypothesis generating and not confirmatory.

Notwithstanding, our large study of patients with true extramedullary involvement treated with ide-cel adds to the existing lacunae of clinical trial information in this space. Our findings suggest a potential role for the incorporation of radiation therapy in EMD, warranting dedicated studies to address this question. Despite responses and outcomes for ide-cel being inferior in patients with EMD, it still represents a valuable option in this context. We hope that our findings serve as a benchmark for future clinical trials to build upon. Identifying effective treatment options for EMD post CAR-T remains an area of active investigation and a deeper understanding of mechanisms of CAR failure in EMD is needed to design better therapies in the future [43, 44]. The encouraging ORR of 83% among patients with EMD at the recommended phase 2 dose combination of teclistamab and talquetamab (REDIRECTT-1) offers excitement as we await dedicated clinical trials for this combination in patients with EMD [7].

In conclusion, our study highlights the significantly adverse prognostic impact of extramedullary disease on outcomes with ide-cel and emphasizes the continuing necessity for improved therapies in this domain.

### Electronic supplementary material

Below is the link to the electronic supplementary material.

Supplementary Material 1

## Data Availability

No datasets were generated or analysed during the current study.
