# Supplementary material for: Impact of extramedullary multiple myeloma on outcomes with idecabtagene vicleucel
Source: J Hematol Oncol. 2024 Jun 6;17:42. doi: 10.1186/s13045-024-01555-4 (PMC11157748; doi:10.1186/s13045-024-01555-4)
Supplement: Supplementary file 1 — Supplementary Material 1 [file 13045_2024_1555_MOESM1_ESM.docx]

Supplementary Material to:

Zanwar, S, Sidana, S, Shune, L, et al. Impact of Extramedullary Multiple Myeloma on Outcomes with Idecabtagene Vicleucel.’

| Table of Contents | | |
| --- | --- | --- |
| Supplementary Table 1 | Predictors of inferior Day 90 ORR | Page 2 |
| Supplementary Table 2. | Univariate analysis of predictors for inferior progression free survival with Ide-cel | Page 3 |
| Supplementary Table 3. | Prognostic markers for patients treated with ide-cel | Page 4 |
| Supplementary Table 4. | Toxicity profile and utilization of mitigation strategies among patients with and without EMD | Page 5 |
| Supplementary Table 5 | Comparison of factors potentially associated with higher stem cell boost requirement | Page 6 |
| Supplementary Figure 1 | Comparison of progression-free and overall survival among patients with extramedullary, paraskeletal and no extramedullary disease. | Page 7 |
| Supplementary Figure 1. | Median Progression-free survival for patients with extramedullary disease with a PET response at day 90 | Page 8 |
| Supplementary Figure 2. | Impact of radiation therapy to extramedullary site on PFS with ide-cel | Page 9 |
| Supplementary Figure 3. | Prognostic markers in patients with EMD infused with ide-cel | Page 10 |

| Supplementary Table 1. Logistic regression analysis for markers associated with Day 90 ORR | | | | |
| --- | --- | --- | --- | --- |
| Parameter | **Univariate Odds Ratio (95% CI)** | **p-value** | **Multivariable Odds Ratio (95% CI)** | **p-value** |
| Extramedullary Disease | **0.24 (0.13-0.4)** | **<0.001** | **0.24 (0.1-0.4)** | **<0.001** |
| Prior BCMA exposure | **0.53 (0.3-0.94)** | **0.03** | **0.48 (0.26-0.9)** | **0.02** |
| Serum Ferritin >400 µg/L at LD | **0.52 (0.31-0.9)** | **0.01** | 0.8 (0.4-1.3) | 0.2 |
| Use of bridging therapy | 1.01 (0.6-1.8) | 0.96 |  |  |
| Response to Bridging Therapy | 1.44 (0.6-4.1) | 0.43 |  |  |
| ECOG PS>1 at LD | 0.53 (0.3-1.1) | 0.09 |  |  |
| Age >70 years | 1.35 (0.8-2.5) | 0.3 |  |  |
| Race | 1.5 (0.9-2.6) | 0.09 |  |  |
| Revised ISS Stage 3 | 1.7 (0.8-4.5) | 0.2 |  |  |
| High-risk Cytogenetics | 0.65 (0.37-1.2) | 0.14 |  |  |
| Plasma cell leukemia | 1.04 (0.3-4.7) | 0.94 |  |  |
| High BMPC (>50%) at LD | 1.2 (0.66-2.3) | 0.53 |  |  |
| Penta-drug refractory | 0.91 (0.54-1.6) | 0.74 |  |  |
| Prior lines of therapy, median | 0.94 (0.85-1.03) | 0.22 |  |  |
| Median cell dose >400 x10^6^/mL | 1.1 (0.65-1.8) | 0.7 |  |  |
| BCMA: B-cell membrane antigen; CRP: c-reactive protein; ECOG: Eastern Cooperative Oncology Group ISS: international staging system; * refractory to bortezomib, carfilzomib, lenalidomide, pomalidomide and CD38 antibody. | | | | |

| Supplementary Table 2. Univariate analysis of predictors for inferior progression free survival with Ide-cel | | |
| --- | --- | --- |
| Parameter | **Hazard Ratio (95% CI)** | **P value** |
| Extramedullary Disease | 2.1 (1.6-2.7) | **<0.001** |
| Revised ISS Stage 3 | 1.5 (1.1-2.1) | **0.02** |
| Use of Bridging Therapy | 1.6 (1.2-2.1) | **0.003** |
| Response to Bridging therapy (ORR) | 1.1 (0.7-1.7) | 0.7 |
| Age >70 years at ide-cel infusion | 0.8 (0.6-1.07) | 0.13 |
| Race (white vs. non-white) | 1.02 (0.8-1.3) | 0.82 |
| Multiple myeloma subtype (intact Immunoglobulin vs. other) | 1.2 (0.9-1.7) | 0.13 |
| Plasma cell leukemia | 2.2 (1.3-3.7) | 0.003 |
| Median lines of therapy prior to Ide-cel | 1.2 (0.9-1.6) | 0.1 |
| High-risk cytogenetics | 1.2 (0.9-1.6) | 0.17 |
| Penta-refractory disease* | 1.3 (1.03-1.7) | **0.03** |
| Prior BCMA exposure | 1.6 (1.2-2.1) | **0.002** |
| ECOG Performance Status >1 at lymphodepletion | 1.8 (1.3-2.5) | **0.0007** |
| Median cell dose >400 x10^6^/mL | 1 (0.8-1.3) | 0.94 |
| Serum ferritin >400 µg/L at lymphodepletion | 1.9 (1.5-2.4) | **<0.0001** |
| CRP >5 mg/L at lymphodepletion | 1.3 (0.99-1.7) | 0.06 |
| High bone marrow plasma cell burden (>50%) at lymphodepletion | 1.4 (1.1-1.9) | 0.01 |
| Lymphodepleting regimen (Flu/Cy vs. others) | 1.3 (0.73-2.2) | 0.4 |
| BCMA: B-cell membrane antigen; CRP: c-reactive protein; ECOG: Eastern Cooperative Oncology Group ISS: international staging system; Flu/Cy: fludarabine/cyclophosphamide * refractory to bortezomib, carfilzomib, lenalidomide, pomalidomide and CD38 antibody. | | |

| Supplementary Table 3. Prognostic markers for overall survival patients treated with ide-cel. | | | | |
| --- | --- | --- | --- | --- |
| Parameter | **Univariate Analysis Hazard Ratio (95% CI)** | **Univariate analysis**  **p-value** | **Multivariable Analysis Hazard Ratio**  **(95% CI), n=217** | **Multivariable Analysis**  **p-value** |
| Extramedullary Disease | 1.6 (1.1-2.4) | **0.007** | 1.01 (0.6-1.7) | 0.95 |
| Revised ISS Stage 3 | 1.9 (1.2-2.9) | **0.003** | **2 (1.1-3.5)** | **0.02** |
| Use of Bridging Therapy | 2 (1.3-3.1) | **0.002** | 1.8 (0.94-3.4) | 0.07 |
| ECOG Performance Status >1 at LD | 2.1 (1.4-3.2) | **0.0004** | 1.8 (0.95-3.4) | 0.07 |
| Serum ferritin >400 µg/L at LD | 2.3 (1.6-3.2) | **<0.0001** | **1.8 (1.1-2.9)** | **0.03** |
| ≥PR at Day 90 | 0.36 (0.24-0.53) | **<0.0001** | **0.3 (0.17-0.51)** | **<0.001** |
| Plasma Cell Leukemia | 2.3 (1.2-4.2) | **0.008** | **2.8 (1.2-6.7)** | **0.02** |
| High BMPC (>50%) at LD | 1.5 (1.1-2.2) | **0.02** | 0.84 (0.5-1.5) | 0.55 |
| CRP >5 mg/L at lymphodepletion | 1.4 (0.99-2) | 0.06 |  |  |
| Age >70 years at ide-cel infusion | 0.84 (0.55-1.2) | 0.4 |  |  |
| Race (white vs. non-white) | 0.9 (0.6-1.3) | 0.5 |  |  |
| Median lines of therapy prior to Ide-cel | 1.05 (0.99-1.1) | 0.09 |  |  |
| High-risk cytogenetics | 1.1 (0.8-1.5) | 0.5 |  |  |
| Penta-refractory disease | 1.3 (0.9-1.8) | 0.17 |  |  |
| Prior BCMA exposure | 1.3 (0.9-1.9) | 0.17 |  |  |
| Lymphodepleting regimen (Flu/Cy vs. others) | 1.3 (0.6-2.6) | 0.5 |  |  |
| BCMA: B-cell membrane antigen; BMPC: bone marrow plasma cells; ECOG: Eastern Cooperative Oncology Groung ISS: international staging system; LD: lymphodepletion | | | | |

| Supplementary Table 4. Toxicity profile and utilization of mitigation strategies among patints with and without EMD | | | |
| --- | --- | --- | --- |
| Parameter | **EMD** | **No EMD** | **P value** |
| Grade ≥3 CRS, n (%) | 2 (2) | 4 (1.5) | 0.58 |
| Grade ≥2 CRS, n (%) | 24 (29) | 59 (22) | 0.23 |
| Grade ≥3 ICANS, n (%) | 1 (1) | 13 (5) | 0.13 |
| Grade ≥2 ICANS, n (%) | 8 (10) | 20 (8) | 0.58 |
| Tocilizumab Use, n (%) | 57 (68) | 187 (70) | 0.7 |
| Steroid use, n(%) | 32 (38) | 83 (31) | 0.23 |
| Anakinra, n(%) | 7 (8) | 15 (6) | 0.38 |
| Infection, n(%) | 36 (43) | 87 (33) | 0.09 |
| Intensive Care Unit Admission, n (%) | 6 (7) | 17 (6) | 0.82 |
| GCSF use, n (%) | 61 (73) | 209 (78) | 0.46 |
| TPO receptor agonist use, n(%) | 13 (15) | 36 (13) | 0.58 |
| Stem Cell Boost, n% | 12 (14) | 11 (4) | **0.001** |
| ANC day 30, median | 1.6 (0.9-2.6) | 1.5 (0.9-3.1) | 0.58 |
| Day 30 Neutropenia, %  Any grade  Grade ≥3 | 69  34 | 67  33 | 0.8  0.84 |
| Hemoglobin day 30, median (IQR) | 8.9 (7.9-10.4) | 9.8 (8.2-10.2) | **0.003** |
| Day 30 Anemia, %  Any grade  Grade ≥3 | 92  26 | 86  19 | 0.1  0.23 |
| Platelet Count day 30, median (IQR) | 59 (23-127) | 64 (26-118) | 0.74 |
| Day 30 Thrombocytopenia, %  Any grade  Grade ≥3 | 91  54 | 87  43 | 0.3  0.09 |
| ANC day 90, median (IQR) | 1.9 (1.2-3.8) | 2.5 (1.6-3.9) | 0.07 |
| Day 90 Neutropenia, %  Any grade  Grade ≥3 | 51  21 | 39  9 | 0.07  **0.009** |
| Hemoglobin day 90, median (IQR) | 9.8 (8.6-11.2) | 10.9 (9.5-12.3) | **0.003** |
| Day 90 Anemia, %  Any grade  Grade ≥3 | 83  9 | 68  8 | **0.01**  0.8 |
| Platelet Count day 90, median (IQR) | 107 (46-204) | 131 (64-182) | 0.4 |
| Day 90 Thrombocytopenia, %  Any grade  Grade ≥3 | 74  30 | 62  19 | 0.05  0.07 |
| ANC: absolute neutrophil count; CRS: cytokine release syndrome; EMD: extramedullary disease; GCSF: granulocyte colonoy stimulating factor; ICANS: immune effector-cell associted neurologic syndrome; TPO: thrombopoietin | | | |

| Supplementary Table 5. Comparison of factors potentially associated with higher stem cell boost requirement | | | |
| --- | --- | --- | --- |
| Parameter | **EMD** | **No EMD** | **P value** |
| Alkylator use in bridging therapy, % | 57 | 63 | 0.47 |
| Bone marrow plasma cell burden, median % | 30 | 28 | 0.66 |
| ASCT rates prior to LD, % | 81 | 82 | 0.9 |
| Grade ≥3 neutropenia immediately prior to LD, % | 9 | 9 | 0.96 |
| Grade ≥3 thrombocytopenia immediately prior to LD, % | 19 | 15 | 0.39 |
| Grade ≥3 anemia immediately prior to LD, % | 18 | 13 | 0.11 |
| LD: lymphodepletion | | | |


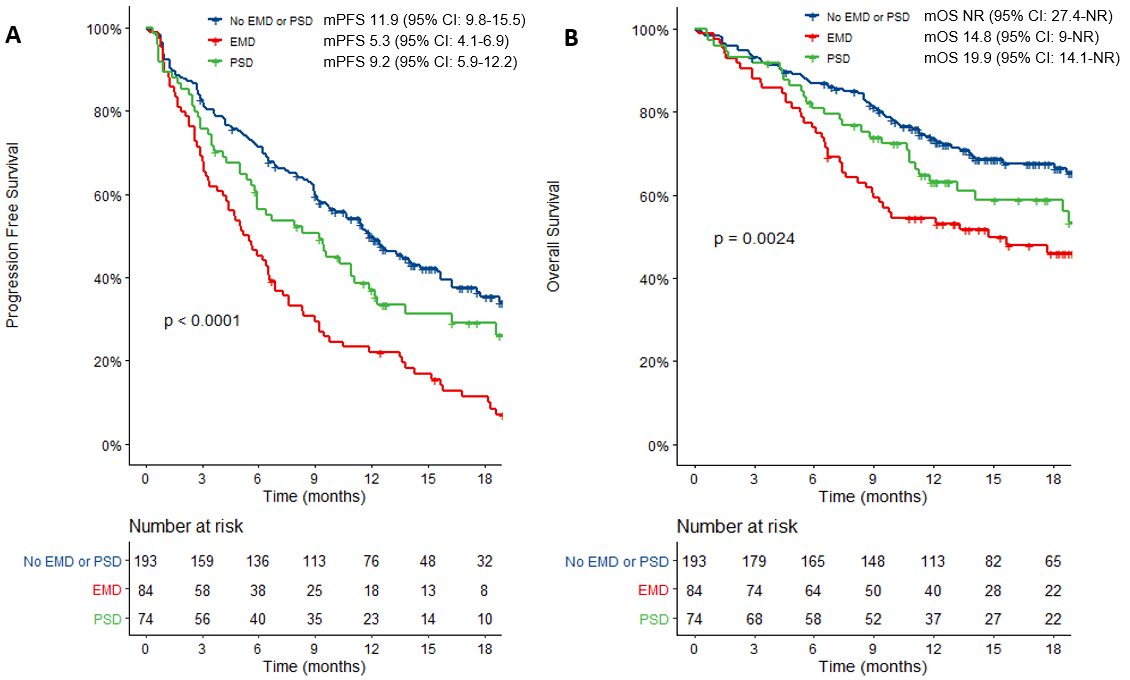


**Supplementary Figure 1.** Extramedullary (EMD) and Paraskeletal Disease (PSD): A. Patients with EMD demonstrated significantly inferior progression-free survival (mPFS) compared to both patients with PSD and non- EMD/PSD cohort. B. Patients with and without EMD demonstrate significantly discrepant median overall survival (mOS), although the difference in OS for EMD vs. PSD did not reach statistical significance.


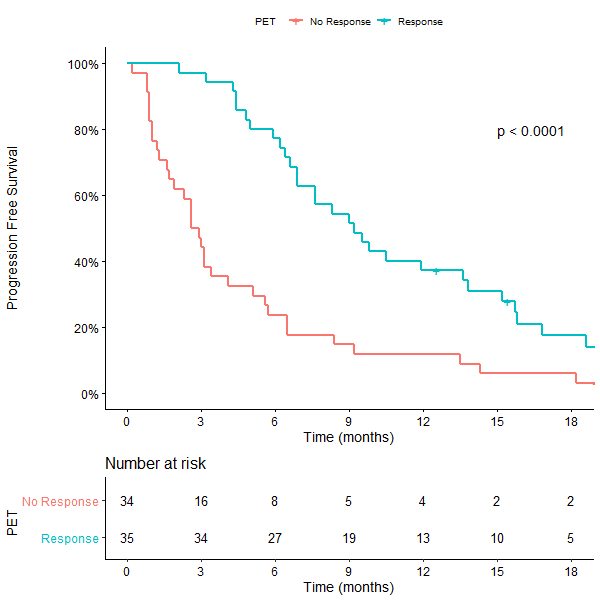


**Supplementary Figure 2.** The PFS was significantly longer for patients with a PET response at day 90 [median PFS 9.2 months (95% CI: 96.9-15.2)] versus non-responders [median PFS 2.7 months (95% CI: 1.9-5.1)] among patients with evidence of extramedullary disease.


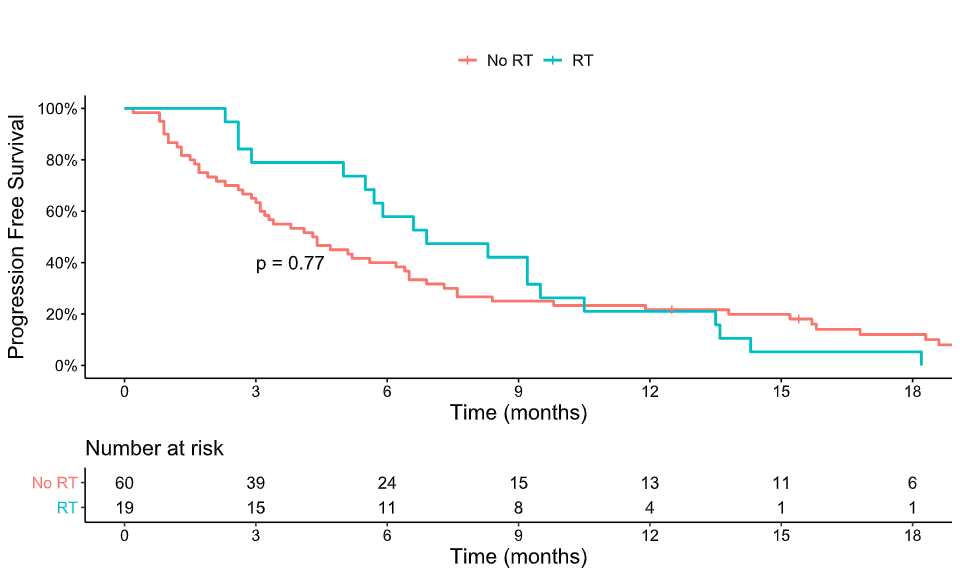


**Supplementary Figure 3**. The median PFS was 6.9 months (95% CI: 5.7-13.6) for patients receiving radiation versus 4.3 months (95% CI: 3.1-6.5) without radiation prior to ide-cel (p=0.77).


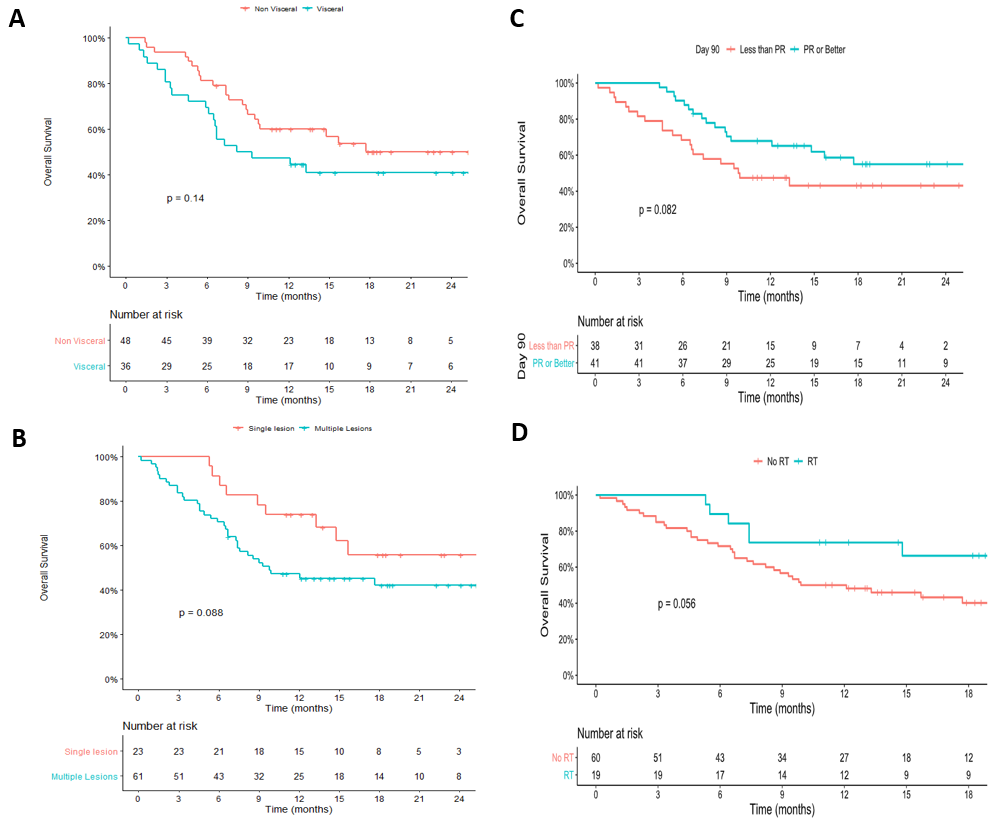


**Supplementary Figure 4:** Prognostic markers for patients with extramedullary disease (EMD) treated with ide-cel. A: Presence of visceral disease [hazard ratio (HR) 1.6 (95% CI: 0.9-2.8), p=0.14)] demonstrated a trend toward inferior OS. B: Multi-site disease (>1 EMD lesion) also demonstrated a trend toward inferior OS [HR 1.9 (95% CI: 0.9-3.9); p=0.09]. C: Achieving an objective response at Day 90 of ide-cel infusion demonstrated a trend toward improved OS [HR 0.57 (0.3-1.08), p=0.08]. D: Radiation therapy for EMD site prior to ide-cel infusion demonstrated a trend toward improved OS [HR 0.44 (95% CI: 0.2-1.04), p=0.056].
